# Growth Arrest-Specific Protein 6 Is Elevated in Endometriosis but Shows Poor Diagnostic Performance

**DOI:** 10.3390/ijms26178348

**Published:** 2025-08-28

**Authors:** Maja Novak Pušić, Robert Marijan, Teja Klančič, Tamara Knific, Helena Ban Frangež, Tea Lanišnik Rižner

**Affiliations:** 1 Institute of Biochemistry and Molecular Genetics, Faculty of Medicine, University of Ljubljana, 1000 Ljubljana, Slovenia; maja.pusic@mf.uni-lj.si (M.N.P.); robert.marijan@mf.uni-lj.si (R.M.);; 2 Department of Obstetrics and Gynecology, University Medical Centre Ljubljana, Šlajmerjeva 3, 1000 Ljubljana, Slovenia; helena.ban@kclj.si

**Keywords:** biomarker discovery, endometriosis, logistic regression, GAS6, CA-125

## Abstract

Growth arrest-specific protein 6 (GAS6) has an important role in regulating the immune system. Recent studies have revealed its association with the pathophysiology of endometriosis and identified GAS6 as one of the hub genes and a biomarker candidate. Endometriosis is a common chronic inflammatory gynaecological disease of women of childbearing age. Due to surgical diagnosis, non-invasive biomarkers are urgently needed. We investigated GAS6 as a candidate biomarker for the diagnosis of endometriosis. Our case–control study included 284 patients and showed that plasma levels of GAS6 are significantly higher in patients with endometriosis compared to control patients. We calculated logistic regression models using GAS6, CA-125, and GAS6 together with CA-125, and added a series of clinical and lifestyle data collected before surgical diagnosis. A CA-125 model and a model including GAS6 and CA-125 showed the highest AUC values of 0.745 ± 0.04, while the model including CA-125, data on sport/recreation before surgery, and dysmenorrhea score reached an AUC of 0.767 ± 0.04. Our results indicate that GAS6 is increased in patients with endometriosis, but it cannot serve as a biomarker candidate.

## 1. Introduction

Growth arrest-specific protein (GAS6) has a wide range of functions. It is associated with tumour development as it regulates cell proliferation, migration, angiogenesis, epithelial to mesenchymal transition, and apoptosis. In addition, GAS6 also modulates the immunological microenvironment of the tumour, regulates the secretion of cytokines and the functions of various immune cells, and thus triggers an immunosuppressive microenvironment of the tumour [1]. GAS6 exerts its effect by binding to the tyrosine kinase receptor AXL, thereby activating various signalling pathways, including JAK/STAT3, PI3K/AKT/mTOR, Grb2/RAS/MEK/ERK1/2, FAK/Src/NF kappa B [1]. Due to its important role in carcinogenesis, GAS6 has been investigated in various types of cancer [2]. Its important role in the regulation of immune system cells suggests that GAS6 may also play a role in benign diseases associated with an impaired immune system, such as systemic lupus erythematosus and venous thromboembolic disease [3], and also in endometriosis [4]. GAS6 was also associated with efferocytosis in ovarian endometriosis lesions compared with eutopic endometrium. Efferocytosis is a process in which apoptotic cells are eliminated by phagocytic cells, primarily macrophages and dendritic cells, and which, when disrupted, contributes to the pathophysiology of endometriosis [3].

Endometriosis is a debilitating chronic inflammatory disease characterised by the presence of endometrial-like stromal and epithelial cells at various sites in the peritoneal cavity [5,6]. It affects 190 million women worldwide and is associated with severe pain and infertility [5,6]. Depending on the location of the ectopic lesions, endometriosis is categorised into three different entities with different pathophysiology: peritoneal, ovarian, and deep endometriosis [7]. The survival of endometrial cells at ectopic sites is enabled by the impaired action of macrophages and other immune cells, and it is increasingly recognised that immune cells play a crucial role in the development and progression of endometriosis [4,8,9].

Common diagnostic procedures include laparoscopy with subsequent histological analysis or imaging procedures such as TVU or MRI [6]. Although ovarian and deep endometriosis can be diagnosed by imaging techniques, laparoscopy is still the gold standard for the diagnosis of peritoneal endometriosis [7]. Due to surgical diagnosis and non-specific symptoms, there is a long delay between the onset of symptoms and surgical confirmation of endometriosis [6]. This delay in diagnosis is associated with disease progression and an increased risk of infertility. Reducing the delay in the diagnosis of endometriosis is therefore an urgent, unmet clinical need that requires the discovery of biomarkers that would enable non-invasive diagnosis.

To our knowledge, there are only a few studies that have investigated GAS6 in endometriosis, and these have all focussed on tissue expression. In this context, GAS6 has emerged as a potential biomarker candidate. Sun et al. showed a significantly higher expression of GAS6 and AXL in ovarian endometriotic tissue compared to normal eutopic endometrium. Using immunohistochemical staining, they detected GAS6 and AXL in epithelial and stromal cells [10]. Additionally, when analysing DNA microarray data comparing endometriotic tissue and eutopic tissue from the same patients, *GAS6* was among the differentially expressed genes and was identified as one of the seven central hub genes—genes that play a crucial role in the development and progression of endometriosis [11]. In addition to its association with efferocytosis in ovarian endometriosis, *GAS6* was significantly upregulated in ectopic versus eutopic tissue, and further univariate analysis showed good diagnostic properties with an AUC > 0.8. The authors constructed a nomogram model that included three biomarker genes, *GAS6*, *ARG2*, and *C3*, with an AUC of 0.97. The upregulation of *GAS6* in ectopic versus eutopic tissue was also confirmed by qRT-PCR and IHC staining. When they analysed single-cell RNA sequencing data from 31 ectopic and 10 eutopic endometrial samples, they found GAS6 in macrophages and also in fibroblasts [3]. Recently, GAS6 was identified in a mouse model of endometriosis when single-cell transcriptomics of endometriosis-associated macrophages was performed. *GAS6* was expressed in tumour-associated-like population that promoted the expression of *COLl1A1* and *TGFB1* in human endometrial stromal cells [4].

Published data showing an important role of GAS6 in the pathophysiology of endometriosis and data reporting its potential as a diagnostic biomarker prompted us to investigate the levels of GAS6 in plasma samples from patients with endometriosis. To our knowledge, this is the first study to investigate GAS6 as a candidate biomarker for the non-invasive diagnosis of endometriosis.

## 2. Results

### 2.1. Clinical Characteristics of Patients

**Figure 1:**
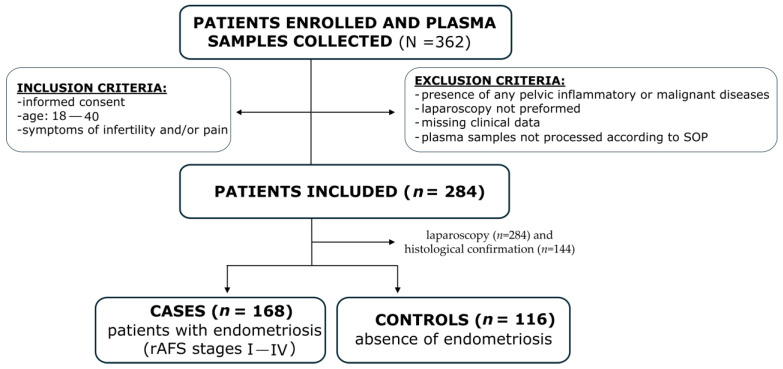
Flow diagram of patient selection.

The cohort consisted of 284 patients, 168 patients with different types of endometriosis and 116 patients with pain and/or infertility (Table 1, Figure 1).

All patients underwent laparoscopy, and the presence of endometriotic lesions was confirmed by histology in 144 patients. The majority of patients had peritoneal endometriosis (*n* = 71), followed by patients with combined ovarian and peritoneal endometriosis (*n* = 38), ovarian endometriosis (*n* = 21), patients with all three types of endometriosis (*n* = 18), ovarian and deep endometriosis (*n* = 9), deep endometriosis (*n* = 6) and peritoneal and deep endometriosis (*n* = 5). There were 86 patients in stage I–II and 82 patients in stage III–IV. The patients from the case and control group did not differ in terms of menstrual phase, use of oral contraceptives and hormone therapy, smoking status, alcohol intake, and recreation in the last two days before laparoscopy.

As expected, there were significant differences between case and control groups of patients in terms of BMI, regularity of menstrual cycle, use of medicines in the last week before surgery, dysmenorrhea score, dyspareunia score, pelvic, abdominal or back pain, frequency of dysmenorrhea, intensity of dysmenorrhea, dyspareunia (general), and dyspareunia (last 3 months) (Table 1, Supplementary Figure S1).

### 2.2. Plasma GAS6 Concentrations Are Increased in Patients with Endometriosis

**Figure 2:**
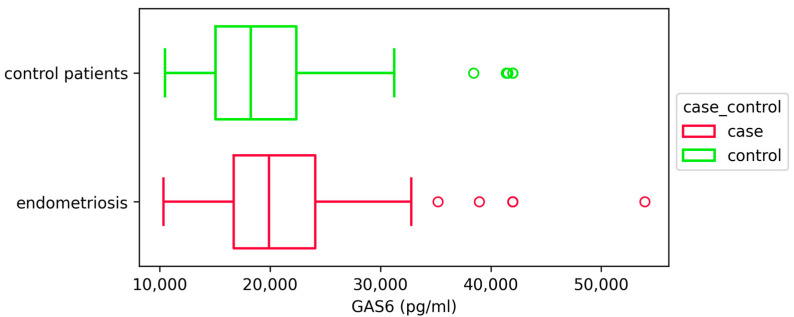
GAS6 concentrations (pg/mL) in patients with and without endometriosis. Data are presented as box plots showing the median and interquartile range; statistical analysis was performed using the Mann–Whitney U test (*p* = 0.015). Patients with endometriosis (*n* = 168), patients without endometriosis (*n* = 116) Outliers are depicted in plot as red and green circles.

We found statistically significantly higher concentrations of GAS6 in plasma samples from patients with different types of endometriosis compared to control patients (*p* = 0.015) (Figure 2, Table 2).

**Figure 3:**
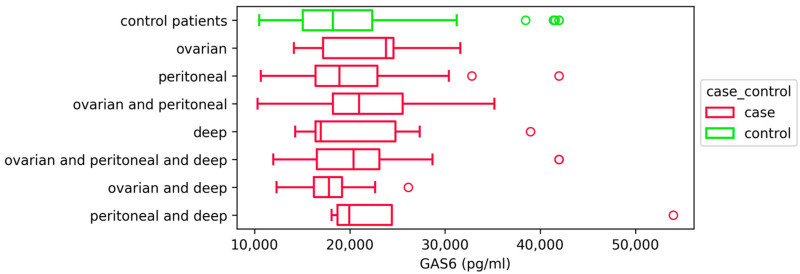
GAS6 concentrations (pg/mL) in patients with different types of endometriosis and controls. Data were presented with box plots showing median and interquartile range; statistical analysis was performed using Kruskal–Wallis H test, *p* = 0.080; ovarian (*n* = 21), peritoneal (*n* = 71), ovarian and peritoneal (*n* = 38), deep endometriosis (*n* = 6), ovarian and peritoneal and deep endometriosis (*n* = 18), ovarian and deep endometriosis (*n* = 9), peritoneal and deep endometriosis (*n* = 5). Outliers are depicted in plot as red and green circles.

We also compared GAS6 concentration between patients with different types of endometriosis and found no statistically significant differences (*p* = 0.080) between these groups. The comparison between the individual types of endometriosis and the control group of patients also showed no significant differences (Figure 3).

**Figure 4:**
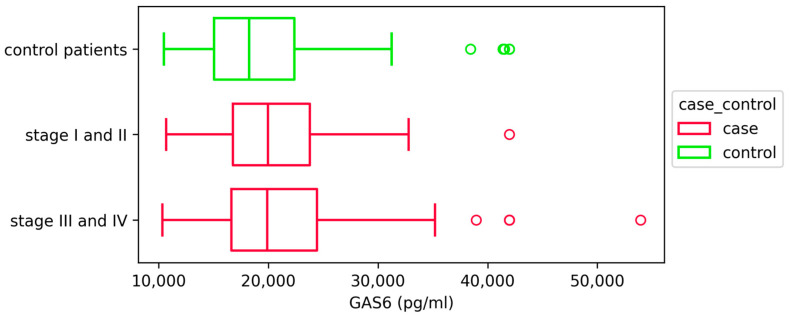
GAS6 concentrations (pg/mL) in patients with different stages of endometriosis and controls. Data are presented as box plots showing median and interquartile range; statistical analysis was performed using Kruskal–Wallis H test, stage I/II (*n* = 86), stage III/IV (*n* = 82), and controls (*n* = 116). Outliers are depicted in plot as red and green circles.

Next, we compared GAS6 concentration between patients with different rAFS stages of endometriosis and also between patients with individual stages of endometriosis and the control group and found no statistically significant differences (*p* = 0.107) (Supplementary Figure S2). When we compared the GAS6 concentration of combined rAFS stages I/II patients and rAFS stages III/IV patients with that of control patients, we also found no statistically significant differences (*p* = 0.051) (Figure 4).

When the patients with endometriosis were stratified according to menstrual phase, use of oral and hormonal therapy, use of medication in the last week, sport/recreation activities two days before surgery (Supplementary Figure S3), and frequency of dysmenorrhea (Supplementary Figure S4), we found no significant differences in the GAS6 values. However, there were statistically significant differences in GAS6 levels in patients stratified by intensity of dysmenorrhea (between the weak and severe pain groups (*p* = 0.032)), dyspareunia in general (between the almost never and quite often groups (*p* = 0.015) and sometimes and quite often groups (*p* = 0.026)) and dyspareunia (in the last 3 months) (between the weak and moderate pain groups (*p* = 0.038)) (Supplementary Figure S4).

The characteristics of GAS6 as a potential biomarker candidate were compared to CA-125. As expected, we found significantly higher levels of CA-125 in patients with endometriosis compared to control patients, higher levels in patients with ovarian endometriosis compared to other types of endometriosis, and higher levels in stages III/IV compared to early stages I/II (Supplementary Figure S5).

### 2.3. Logistic Regression Models Including GAS6 and CA-125

**Figure 5:**
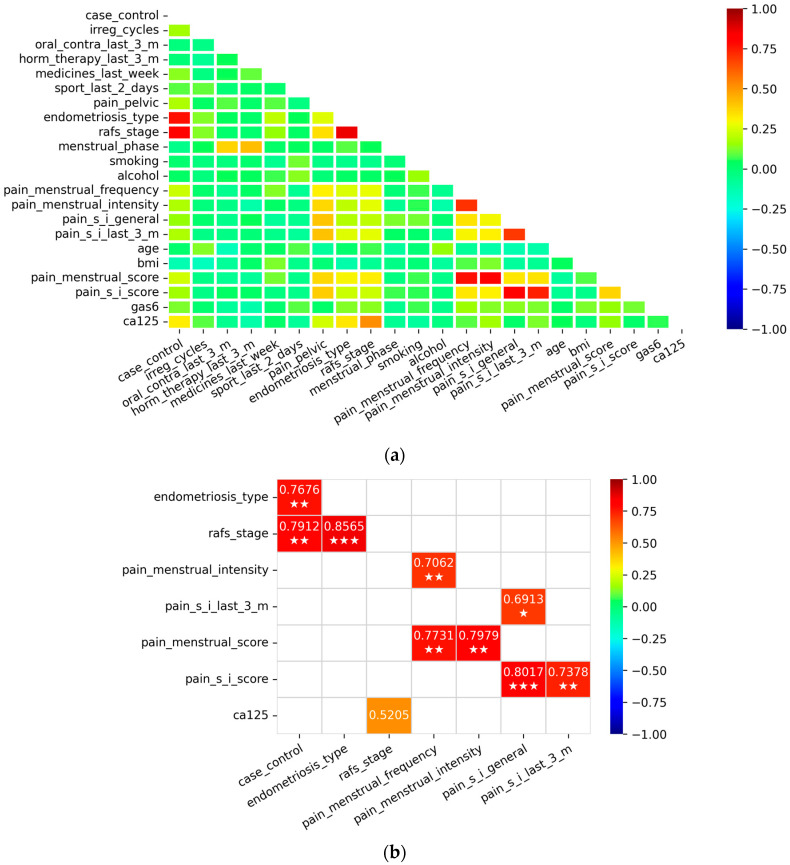
Pearson’s correlation matrix between dataset variables: (**a**) Pearson’s correlation matrix used for multicollinearity estimation. Y-scale showing strength and direction of an association between variables; (**b**) identified correlated variables inside correlation matrix. One white star represents association cutoff above 0.60, two white stars represents association cutoff above 0.70, and three white stars represents association cutoff above 0.80 value. Y-scale showing strength and direction of an association between variables.

We continued with the construction of logistic regression diagnostic models. First, we estimated the multicollinearity between the predictors using the Pearson correlation factor and the variance inflation factor (VIF) (Figure 5). The aim was to prevent the identified correlated predictors from being part of the same multivariable logistic regression model.

We attempted to identify predictors capable of discriminating between case and control patients. Each model was iterated 300 times, repeatedly splitting the training/test data. The ratio between training and test groups was 0.66 versus 0.33. All iterations were case/control stratified.

In addition to GAS6 and CA-125, we used another 17 clinical and lifestyle data that were available before laparoscopy: age, BMI, menstrual phase, regularity of menstrual cycle, oral contraception in the last three months, hormone therapy in the last three months, medication in the last week, dysmenorrhoea score, pelvic, abdominal or back pain, frequency of dysmenorrhoea, intensity of dysmenorrhoea, dyspareunia (general) and dyspareunia in the last three months, dyspareunia score, sport/recreation activities in the last two days (before the operation), smoking status, and alcohol consumption.

Since we found a statistically significantly higher concentration of GAS6 in plasma samples from patients with different types of endometriosis compared to control patients, we expected that GAS6 alone and in combination with CA-125 and clinical data would be a significant predictor in logistic regression models with case/control discrimination.

Single-variable GAS6 logistic regression models had poor discriminatory power (GAS6 mean *p*-value of 0.163 ± 0.16) with a mean AUC of 0.583 ± 0.05, a mean sensitivity of 96 ± 6% and a mean specificity of 5 ± 7%. The GAS6 random forest models had a mean AUC of 0.536 ± 0.05 with a mean sensitivity of 63 ± 7% and a mean specificity of 46 ± 8% (Table 3, Supplementary Figure S6).

**Figure 6:**
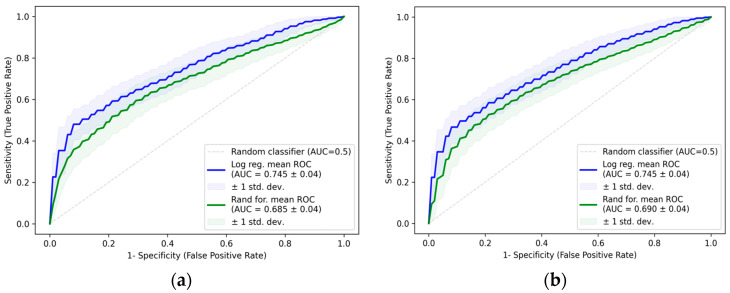
Receiver operating characteristic (ROC) curves for logistic regression and random forest models: (**a**) CA-125 logistic regression and random forest models; (**b**) combined GAS6 and CA-125 logistic regression and random forest models.

The single-variable logistic regression model with CA-125 had a mean AUC of 0.745 ± 0.04, the mean AIC was 217.74 ± 6.81, the mean sensitivity was 66 ± 7%, and the mean specificity was 67 ± 7% (Table 3, Figure 6). This model had the highest mean AUC of all calculated one-variable models, followed by models with a significantly lower ability to discriminate between case and control patients (dysmenorrhea score with a mean AUC of 0.638 ± 0.05, dysmenorrhea—frequency with a mean AUC of 0.628 ± 0.05 (Supplementary Table S1).

The average AUC value of the combined logistic regression model of GAS6 and CA-125 was comparable to the single CA-125 model, with an AUC value of 0.745 ± 0.04 and an average AIC value of 218.52 ± 6.81. The average sensitivity did not improve, while the average specificity improved slightly compared to the single CA-125 model. The random forest models improved slightly compared to the random forest models of a single CA-125 model. The data can be seen in Table 3 and Figure 6.

**Figure 7:**
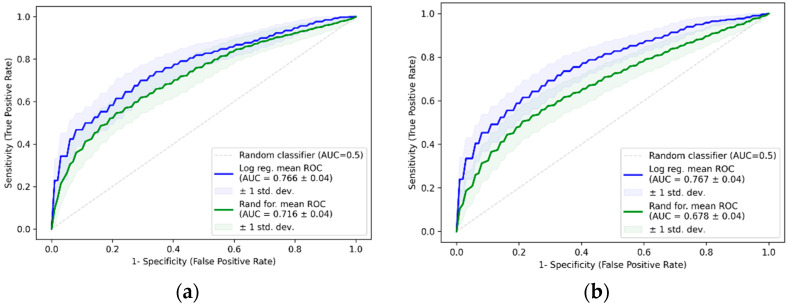
Receiver operating characteristic (ROC) curves for combined logistic regression and random forest models: (**a**) combined CA-125 and frequency of dysmenorrhea logistic regression and random forest models; (**b**) combined CA-125, sport/recreation in the last two days before surgery, and dysmenorrhea score logistic regression and random forest models.

The best model with GAS6 as a fixed predictor (without CA-125) included the frequency of dysmenorrhea and showed a mean AUC of 0.641 ± 0.05, which is higher than the GAS6 model with an AUC of 0.583 ± 0.05. The best two-variable model was CA-125 in combination with the frequency of dysmenorrhea, with an average AUC of 0.766 ± 0.04, which was again slightly higher than the CA-125 model with 0.745 ± 0.04 (Figure 7). The best three-variable model included CA-125, exercise/recreation in the last two days before surgery, and the dysmenorrhea score, with an average AUC of 0.767 ± 0.04 (Figure 7).

The mean AUC value of the 3-variable models did not differ significantly when CA-125 was part of the predictor combination. We could not single out any variables (such as sport/recreation in the last two days before surgery) because the differences in AUC values were not that significant.

**Figure 8:**
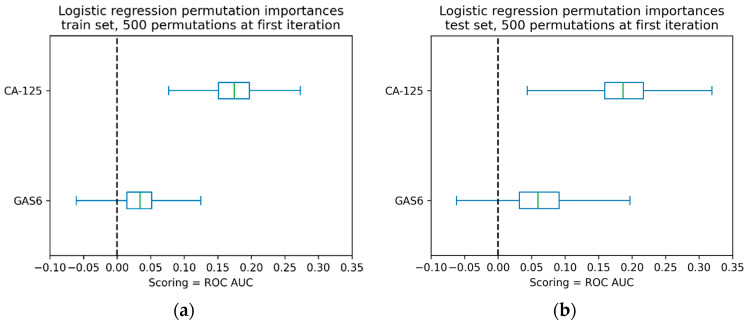
AUC predictor importance of the combined GAS6 and CA-125 logistic regression model using the permutation feature importance method. Data for (**a**) training and (**b**) test set are shown.

Finally, we estimated the AUC predictor significance of the combined logistic regression model of GAS6 and CA-125 using the permutation method for feature significance (Figure 8). We can see that CA-125 had a significantly larger effect on the AUC value (500 permutations at the first model iteration) than GAS6.

## 3. Discussion

This is the first study to investigate GAS6 as a potential diagnostic biomarker for endometriosis. The hypothesis that GAS6 can be found in higher concentrations in blood samples from patients with endometriosis compared to control patients was confirmed. We found statistically higher levels of GAS6 in cases compared to the control groups. There were no differences between the patient groups stratified according to different types and stages of endometriosis, menstrual phase, use of oral and hormonal therapies, use of medication in the last week, sports activities two days before surgery, and frequency of dysmenorrhea. However, we found statistically significant differences in GAS6 levels stratified by intensity of dysmenorrhea, dyspareunia in general, and dyspareunia (last 3 months). The logistic regression models that included GAS6 showed poor discriminatory power capabilities (AUC 0.583). As expected, the model with GAS6 and CA-125 had better properties (AUC 0.745) but did not achieve a higher AUC than CA-125 alone. The inclusion of the variable frequency of dysmenorrhea in the CA-125 model increased the AUC to 0.766 with a sensitivity of 74% and a specificity of 63%. The model that included CA-125, the dysmenorrhea score, and the additional variable sport/recreation before surgery showed a similar AUC value of 0.767 with a sensitivity of 73% and a specificity of 66%.

It is well known and was also reported in our previous study that CA-125 only better discriminates stage III-IV patients from control patients [12]. Nevertheless, CA-125 is still the only biomarker available in the clinical setting. Therefore, the models based on CA-125, clinical data, and pre-laparoscopy lifestyle data can be easily translated to clinical application if sensitivity and specificity are high enough. Several models, including CA-125, have been reported. Combinations between CA-125 and urinary levels of vitamin D-binding protein, CA-125 in combination with duration of menstruation, and endometrial leucocyte levels even met the criteria for rule in triage test for pelvic endometriosis [13]. We have previously reported logistic regression models including serum CA-125, BMI, cyst pathology, dysmenorrhea, or dyspareunia with good diagnostic properties and AUC values of 0.836 and 0.819, respectively [14,15]. However, none of the CA-125 models presented so far included the exercise variable.

Different types of endometriosis represent different entities with different pathogenesis. Published studies have identified GAS6 as an important hub gene [3,11] associated with the pathophysiology of ovarian endometriosis, with significantly higher expression in ectopic versus eutopic tissue and with good diagnostic properties (AUC > 0.8) [3]. *GAS6* was also upregulated in ectopic tissue derived from peritoneal, ovarian, and deep endometriosis compared to eutopic endometrium (https://endometdb.utu.fi/gene_analysis/, accessed on 31 July 2025. Supplementary Figure S7 [16]). Consistent with previous studies showing expression of GAS6 in macrophages, particularly in tumour-associated-like populations [4], GAS6 may also be associated with peritoneal macrophages, which are known to play a role in the development of endometriotic lesions [17]. Therefore, we hypothesised that GAS6 levels are elevated in patients with different types of endometriosis. Although we detected elevated levels in the combined cohort of patients with endometriosis, we found no differences between the individual types of endometriosis and the control group of patients. It is possible that GAS6 plays a role in the pathophysiology of the different types of endometriosis, but the plasma concentrations do not reflect the tissue concentrations.

There are several reasons why GAS6 might be elevated in tissue but not in plasma. It is possible that it is primarily present locally in the endometriotic lesions, but is diluted upon release into plasma. In addition, GAS6 has been shown to bind to the soluble form of its receptor, sAxl, which prevents stimulation of the TAM receptor and suggests more localised effect [18]. Plasma levels are known to reflect systemic changes as plasma circulates in the body, so elevated GAS6 levels may indicate other systemic processes rather than specific endometriosis.

Although our results do not support the use of plasma GAS6 as a diagnostic biomarker for endometriosis, we observed that GAS6 levels are associated with pain symptoms (dysmenorrhoea and dyspareunia). This suggests that GAS6 may contribute to the pathophysiology of the disease rather than the diagnosis. GAS6 regulates the immune response via TAM receptors and has a dual function by either enhancing or suppressing proinflammatory signalling depending on the disease and microenvironment [19,20]. In addition, TAM receptors play an important role in modulating neuroinflammation and pain by regulating microglial activation and promoting the resolution of inflammation [21]. Importantly, GAS6 has been proposed as a broad regulator of the innate immune response, and possibly reflecting general inflammatory activity rather than disease-specific pathology [22]. GAS6 also increases endothelial cell activation in response to inflammatory stimuli and accelerates the circulation of platelets and leukocytes in blood [20], which may be indicative of chronic systemic inflammation. Future studies need to clarify whether GAS6 contributes directly to the mechanism of endometriosis-associated pain or primarily reflects systemic inflammation.

This study has several strengths: It includes a population with a relatively large number of patients with different types of endometriosis (*n* = 168) and a control group of symptomatic patients (*n* = 116). As we know that pre-analytical errors can influence the final results, blood samples were collected according to a strict SOP [23]. The included patients were well characterised by clinical and lifestyle data. Experimental data were analysed using logistic regression by performing 300 stratified train/test iterations. We report the mean AUC with SD, AIC, sensitivity, and specificity for the models generated.

The weaknesses of this study include its retrospective design, which was based on previously collected clinical data and samples, and the lack of an external, multicentre validation cohort. In addition, the study only analysed the GAS6 protein and the GAS6 protein in combination with CA-125. As endometriosis is a complex pathology, it is clear that individual biomarkers cannot achieve the sensitivity and specificity required for clinical application. Better performance is expected from algorithms that combine different biomarkers with clinical and/or lifestyle data, which was also demonstrated in our study.

In contrast to the targeted approach used in this study, the search for endometriosis biomarkers increasingly relies on high-throughput technologies such as RNA-seq, proteomics, lipidomics, and metabolomics [24]. Using these approaches, a variety of molecules, including proteins, miRNAs, lncRNAs, and metabolites, have been identified as potential non-invasive biomarker candidates in plasma, serum, and other biological fluids such as saliva, urine, and menstrual blood [25,26,27,28]. More recently, single-cell RNA sequencing of peripheral blood mononuclear cells (PBMCs) [29] and menstrual blood samples [30] has been used to detect disease-specific molecular signatures. In parallel, machine learning techniques are used to integrate clinical information with large omics datasets to identify novel biomarker candidates [31].

From the results of this study, we conclude that GAS6 is not a biomarker candidate for endometriosis. However, our results suggest that GAS6 may be associated with pain symptoms, indicating a possible role in the pathophysiology of the disease, which should be further investigated in future studies. In addition, our study has shown that CA-125 in combination with the variable frequency of dysmenorrhea achieves better diagnostic properties than CA-125 alone.

## 4. Materials and Methods

### 4.1. Study Design and Patient Selection

This study was designed as a retrospective case–control study and was conducted with the approval of the Medical Ethics Committee of the Republic of Slovenia (No. 120-12772016-2 and No 0120-049/2016-4). Written informed consent was obtained from all participants included in the study.

A total of 362 patients were included in the study based on the following inclusion criteria: signed informed consent, reproductive age (18–40 years), problems with infertility, and/or symptoms suggestive of endometriosis. The exclusion criteria were the presence of pelvic inflammatory or malignant disease, laparoscopic surgery not performed, plasma samples not processed according to the SOP, and missing clinical data. After applying the exclusion criteria, the final study cohort comprised 284 patients. All patients underwent laparoscopy and were characterised by the presence of endometriosis (cases, *n* = 168) and the absence of endometriosis (controls, *n* = 116). Patients with endometriosis were categorised into stages I–IV (from minimal/mild to moderate/severe) according to the revised American Fertility Society (rAFS) score (Figure 1). Potential confounders such as menstrual cycle phase, hormonal therapy, or inflammation markers were not part of the inclusion or exclusion criteria for patient selection.

### 4.2. Sample and Data Collection

All patients who met the inclusion criteria completed a comprehensive questionnaire developed by our research group [15] on their medical history, stress levels, medication use, diet, lifestyle habits, and the nature of their pain (dysmenorrhea, dyspareunia, or chronic pain) using a validated visual analogue scale [32]. A gynaecologist completed a further questionnaire with gynaecological and clinical information about each patient, including length and regularity of menstrual cycle, use of peroral contraception and/or hormonal therapy in the past and in the last 3 months before surgery, use of medication 1 week before surgery, type and reason for surgery, type of endometriosis, rAFS stage, colour of lesions, histological confirmation of endometriosis, menstrual phase determined by ultrasound and additional pathological observations.

The blood samples were taken at the Department of Obstetrics and Gynaecology at the University Medical Centre Ljubljana in Slovenia, one day to one week before the operation according to a strict standard procedure [23]. In brief, two tubes of blood samples (4 mL each) were obtained by venipuncture from the median cubital vein. To obtain the plasma, the blood was placed in EDTA-containing BD Vacutainer tubes (#368861, Becton Dickinson and Company, Franklin Lakes, NJ, USA). The tubes were inverted 10 times to ensure sufficient mixing with the anticoagulant and immediately stored at +4 °C. BD Vacutainer tubes with a gel separator and clot activator (#369032, Becton Dickinson and Company, Franklin Lakes, NJ, USA) were used to collect the serum. All samples were processed within one hour of collection. The collected blood samples were centrifuged at 2500× *g* (plasma) and 1400× *g* (serum) for 10 min at +4 °C. The plasma and serum were then collected, aliquoted, and stored at −80 °C until further analysis.

### 4.3. ELISA and ECLIA

GAS6 was measured in 1:5 diluted plasma samples using a commercial GAS6 Human Enzyme-Linked Immunosorbent Assay (ELISA) kit (Cat.No.: B0AB04090J00065, lot number: A9559A332A631, lot number: I9859A332A631, and lot number: J9859A332A631, Abnova Corporation, Taipei City, Taiwan) according to the manufacturer’s instructions.

Serum samples were analysed using a clinically validated electrochemiluminescent immunoassay for CA-125 on an immunoassay analyser (Cobas e411, Roche Diagnostics GmbH, Mannheim, Germany). The data on CA-125 values have been reported previously [12,14].

### 4.4. Statistical Analyses

To identify significant statistical differences between the variables in the dataset, we used Python 3.12.3, the Python module statsmodels 0.14.4, which provides classes and functions for estimating different statistical models, SciPy 1.15.1—basic algorithms for scientific computing in Python, and Scikit-learn—a library for machine learning in Python. We manually checked the detected outliers and, as they were measured correctly, left them in the dataset. From the final cohort of 284 patients, clinical variables with less than 10% of missing values per patient were imputed with the median value of available values.

We performed a descriptive analysis of the dataset. We estimated the data distributions using the Shapiro–Wilk test. As the data were not normally distributed, we used non-parametric tests.

We assessed statistical differences by using Fisher’s exact tests for categorical variables and Mann–Whithey U tests for the comparison of GAS6 protein concentration values between the case and control groups of patients and for other continuous variables. We used the Kruskal–Wallis H test (one-way ANOVA on ranks) for comparisons between multiple groups, followed by a post hoc Dunn test with Holm–Sidak correction method to adjust *p* values for multiple comparisons.

We calculated logistic regression models using the Python library statsmodels. Each predictor combination was iterated 300 times, repeatedly splitting the training/test data. The ratio between training and test groups was 0.66 versus 0.33. All iterations were stratified by cases/controls. We used the average AUC, average AIC, average sensitivity, and average specificity over 300 calculated iterations for each predictor combination. We calculated the best 1-variable, 2-variable, and 3-variable logistic regression models (Supplementary Table S1).

## Figures and Tables

**Table 1:** Clinical characteristics of the study participants.

Parameter	Subgroup	Cases, *n* = 168 Mean ± SD/*n* (%)	Controls, *n* = 116 Mean ± SD/*n* (%)	*p*-Value *
age (years)	-	30.51 ± 4.11	30.38 ± 4.88	ns
BMI (kg/m^2^)	-	22.82 ± 3.83	24.01 ± 4.64	*p* < 0.05
menstrual phase	OHC	17 (0.10)	14 (0.12)	ns
	proliferative	82 (0.49)	51 (0.44)	
	secretory	69 (0.41)	51 (0.44)	
type of endometriosis	0_no_endometriosis	0 (0.00)	116 (1.0)	0 = control
	1_ovarian	21 (0.12)	0 (0.0)	
	2_peritoneal	71 (0.42)	0 (0.0)	
	3_ovarian_and_peritoneal	38 (0.23)	0 (0.0)	
	4_deep	6 (0.04)	0 (0.0)	
	5_ovarian_and_peritoneal_and_deep	18 (0.11)	0 (0.0)	
	6_ovarian_and_deep	9 (0.05)	0 (0.0)	
	7_peritoneal_and_deep	5 (0.03)	0 (0.0)	
rAFS stage	0_no_endometriosis	0 (0.00)	116 (1.0)	0 = control
	I	61 (0.36)	0 (0.0)	
	II	25 (0.15)	0 (0.0)	
	III	52 (0.31)	0 (0.0)	
	IV	30 (0.18)	0 (0.0)	
regularity of menstrual cycle	no	4 (0.02)	12 (0.1)	*p* < 0.05
	yes	164 (0.98)	104 (0.9)	
oral contraception last 3 months	no	155 (0.92)	106 (0.91)	ns
	yes	13 (0.08)	10 (0.09)	
hormonal therapy last 3 months	no	143 (0.85)	98 (0.84)	ns
	yes	25 (0.15)	18 (0.16)	
medicines—last week	no	107 (0.64)	89 (0.77)	*p* < 0.05
	yes	61 (0.36)	27 (0.23)	
dysmenorrhea score	visual analogue scale (1–10)	6.00 ± 2.50	4.73 ± 2.67	*p* < 0.05
dyspareunia score	visual analogue scale (1–10)	2.70 ± 2.40	1.91 ± 2.02	*p* < 0.05
dysmenorrhea—frequency	1_never	0 (0.00)	2 (0.02)	*p* < 0.05
	2_almost_never	11 (0.07)	21 (0.18)	
	3_sometimes	34 (0.20)	22 (0.19)	
	4_quite_often	25 (0.15)	31 (0.27)	
	5_very_often	98 (0.58)	40 (0.34)	
dysmenorrhea—intensity	0_no_pain	16 (0.10)	22 (0.19)	*p* < 0.05
	1_slight_pain	62 (0.37)	55 (0.47)	
	2_medium_pain	60 (0.36)	24 (0.21)	
	3_strong_pain	30 (0.18)	15 (0.13)	
dyspareunia (general)	1_never	43 (0.26)	38 (0.33)	*p* < 0.05
	2_almost_never	39 (0.23)	31 (0.27)	
	3_sometimes	52 (0.31)	37 (0.32)	
	4_quite_often	17 (0.10)	7 (0.06)	
	5_very_often	17 (0.10)	3 (0.03)	
dyspareunia (last 3 months)	0_no_pain	56 (0.33)	58 (0.50)	*p* < 0.05
	1_slight_pain	72 (0.43)	42 (0.36)	
	2_medium_pain	36 (0.21)	15 (0.13)	
	3_strong_pain	4 (0.02)	1 (0.01)	
pelvic, abdominal or back pain	no	106 (0.63)	93 (0.8)	*p* < 0.05
	yes	62 (0.37)	23 (0.2)	
smoking status	1_non_smoker	105 (0.62)	73 (0.63)	ns
	2_smoker	42 (0.25)	28 (0.24)	
	3_smoker_occas_week	6 (0.04)	3 (0.03)	
	4_smoker_occas_month	2 (0.01)	4 (0.03)	
	5_smoker_former	13 (0.08)	8 (0.07)	
alcohol	1_never	47 (0.28)	30 (0.26)	ns
	2_rarely	102 (0.61)	76 (0.66)	
	3_once_a_week	9 (0.05)	7 (0.06)	
	4_2_to_3_times_a_week	8 (0.05)	2 (0.02)	
	5_more_than_3_times_a_week	2 (0.01)	1 (0.01)	
sport/recreation—last 2 days (before surgery)	no	109 (0.65)	85 (0.73)	ns
	yes	59 (0.35)	31 (0.27)	

* Fisher’s exact tests for categorical variables, Mann–Whitney U tests for continuous variables, Kruskal–Wallis H tests for multiple group comparisons (ns = statistically not significant, *p*-value > 0.05).

**Table 2:** GAS6 and CA-125 concentrations in patients with and without endometriosis.

Parameter	Cases, *n* = 168 Mean ± SD/*n* (%)	Controls, *n* = 116 Mean ± SD/*n* (%)	*p*-Value *
GAS6 (pg/mL)	21,056.09 ± 6664.89	19,499.93 ± 6465.49	*p* < 0.05
CA-125 (U/mL)	50.33 ± 61.46	17.50 ± 13.33	*p* < 0.05

* Mann–Whitney U tests for continuous variables (ns = statistically not significant, *p*-value > 0.05).

**Table 3:** Characteristics of logistic regression and random forest models. Average AUC, average AIC, average sensitivity, and average specificity for GAS6, CA-125, and multivariable GAS6 and CA-125 logistic regression and random forest models.

Method	Predictor	Avg AUC ± sd	Avg AIC ± sd	Avg Sensitivity ± sd (%)	Avg Specificity ± sd (%)
Log reg.	GAS6	0.583 ± 0.05	258.17 ± 2.09	96 ± 6	5 ± 7
Rand for.	GAS6	0.536 ± 0.05	/	63 ± 7	46 ± 8
Log reg.	CA-125	0.745 ± 0.04	217.74 ± 6.81	66 ± 7	67 ± 7
Rand for.	CA-125	0.685 ± 0.04	/	69 ± 6	55 ± 8
Log reg.	GAS6 + CA-125	0.745 ± 0.04	218.52 ± 6.81	66 ± 7	68 ± 7
Rand for.	GAS6 + CA-125	0.690 ± 0.04	/	70 ± 6	56 ± 8
Log reg.	CA-125 + dysmenorrhea frequency	0.766 ± 0.04	214.37 ± 7.18	74 ± 7	63 ± 9
Log reg.	CA-125 + dysmenorrhea score + sport last two days before surgery	0.767 ± 0.04	214.08 ± 7.05	73 ± 7	66 ± 7

## Data Availability

The raw data supporting the conclusions of this article will be made available by the authors upon request.

## Supplementary Materials

The following supporting information can be downloaded at: https://www.mdpi.com/article/10.3390/ijms26178348/s1.

## Abbreviations

The following abbreviations are used in this manuscript:

AIC	Akaike’s Information Criterion
AKT	Proteinase kinase B
ARG2	Arginase 2
AUC	Area under the curve
AXL	AXL receptor tyrosine kinase
BMI	Body mass index
C3	Complement component 3
CA-125	Cancer antigen 125
ECLIA	Electrochemiluminescence immunoassay
EDTA	Ethylenediamine tetraacetic acid
ELISA	Enzyme-linked immunosorbent assay
ERK1/2	Extracellular signal-regulated kinase 1/2
FAK	Focal adhesion kinase
Grb2	Growth factor receptor-bound protein 2
GAS6	Growth arrest-specific protein 6
IHC	Immunohistochemistry
JAK/STAT3	Janus kinase/signal transducer and activator of transcription 3 pathway
MEK	Mitogen-activated protein kinase kinase
MRI	Magnetic resonance imaging
mTOR	Mechanistic target of rapamycin
NF kappa B	Nuclear factor kappa-light-chain-enhancer of activated B cells
PIK3	Phosphatidylinositol 3-kinase
qRT-PCR	Quantitative real-time polymerase chain reaction
rAFS	revised American Fertility Society score
RAS	Rat sarcoma
ROC	Receiver operating characteristic curve
SOP	Standard operating procedure
Src	Proto-oncogene tyrosine-protein kinase
TGFB1	Transforming growth factor beta 1
TVU	Transvaginal ultrasound
VIF	Variance inflation factor
