# Supplementary material for: Growth Arrest-Specific Protein 6 Is Elevated in Endometriosis but Shows Poor Diagnostic Performance
Source: Int J Mol Sci. 2025 Aug 28;26(17):8348. doi: 10.3390/ijms26178348 (PMC12427796; doi:10.3390/ijms26178348)
Supplement: Supplementary file 1 [file ijms-26-08348-s001.zip › ijms-3822696-supplementary.pdf]

Growth arrest-specific protein 6 is elevated in patients with endometriosis compared to control patients but shows poor characteristics as biomarker candidate

Maja Pušić Novak [1#], Robert Marijan [2#], Teja Klančič [3], Tamara Knific [4], Helena Ban-Frangež[5] and Tea Lanišnik Rižner [6*]

Supplementary data

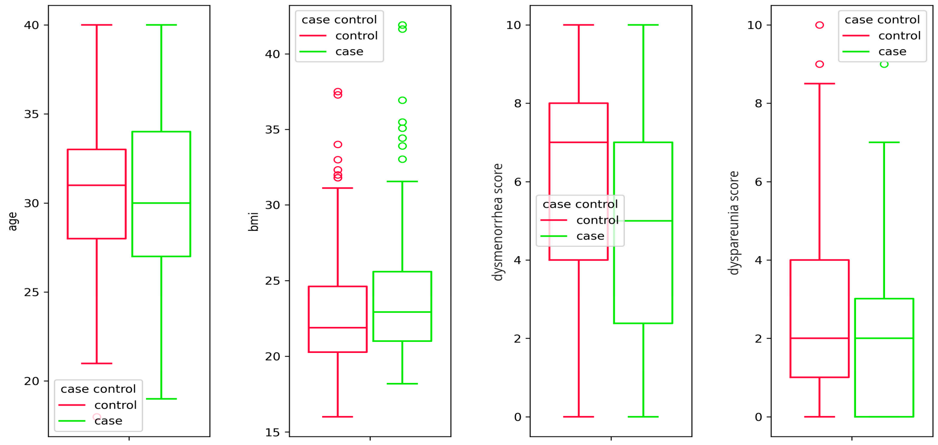

Supplementary Figure S1. Comparison of age, BMI, dysmenorrhoea score and dyspareunia score between patients with and without endometriosis. Data are shown as boxplots with median and interquartile range; statistical analysis was performed using the Mann–Whitney U test. The score for dysmenorrhoea and dyspareunia was determined using a visual analogue scale from 1 to 10. Outliers are shown as circles.

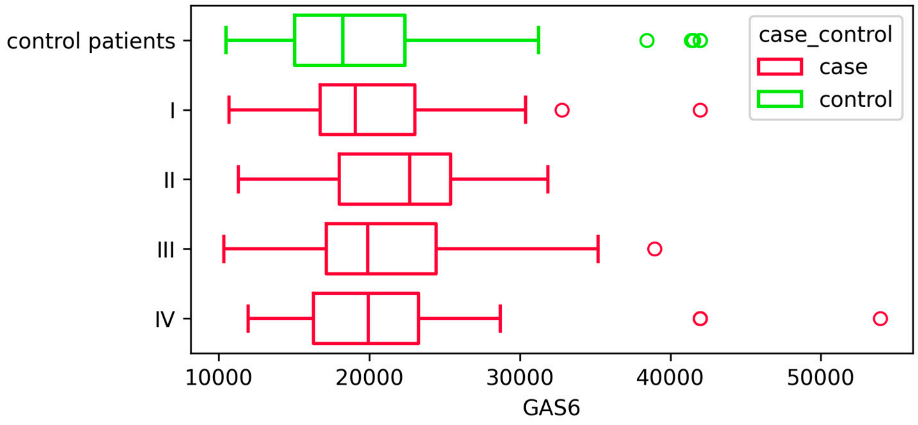

Supplementary Figure S2. Boxplot showing the comparison between the GAS6 concentration (pg/ml) of patients with different rAFS stages of endometriosis (case) and control patients (control). (Kruskal–Wallis H-test, p = 0.1068). Post hoc tests were not performed. Outliers are shown as circles.

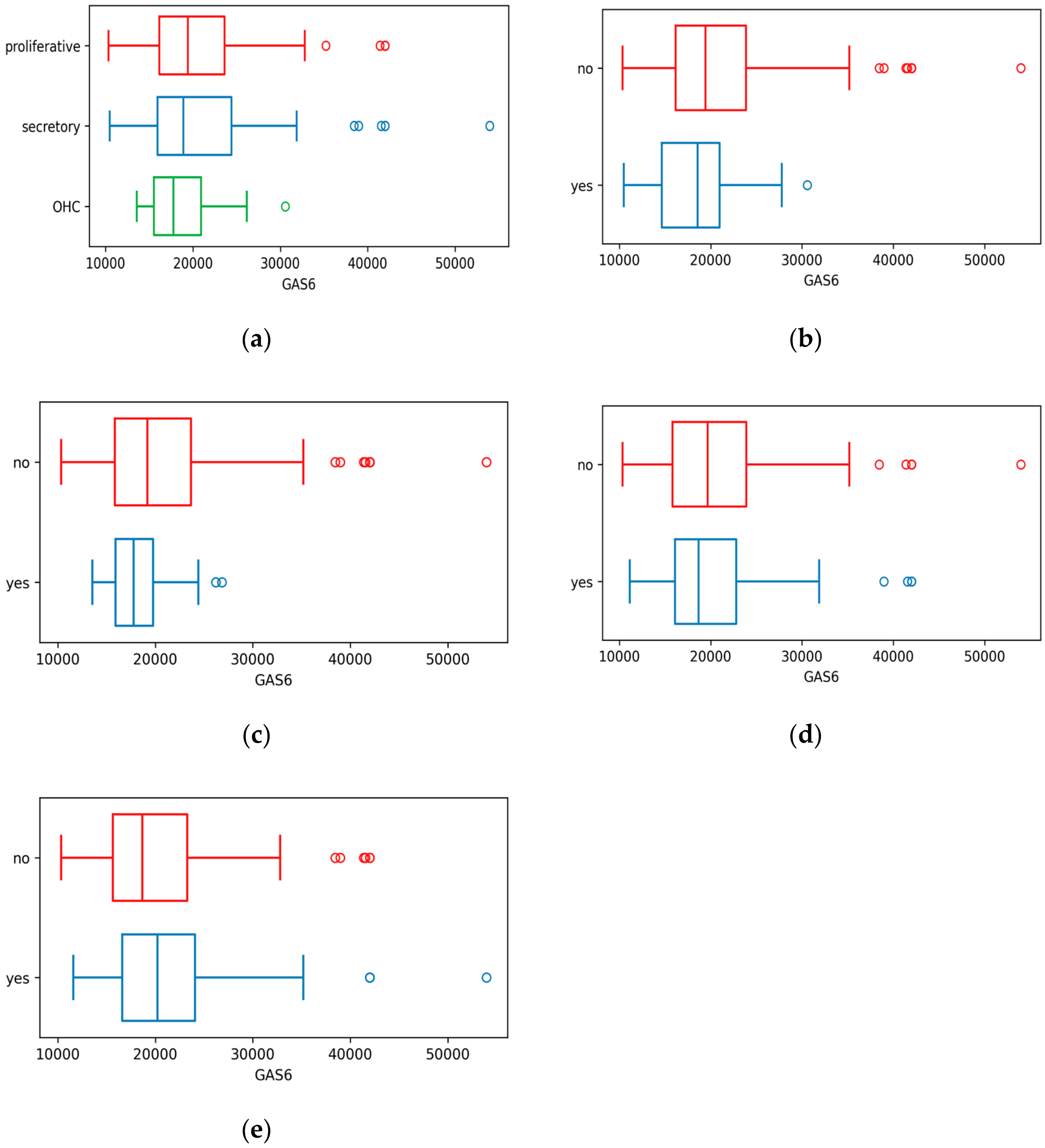

Supplementary Figure S3. Comparison of GAS6 concentration (pg/ml) stratified by menstrual phase, hormone therapy and oral contraception in the last three months before surgery and sport/recreation in the last two days before surgery. Boxplot showing GAS6 concentrations according to (a) menstrual phase of the patient (Kruskal–Wallis H-test, $p = 0.3784$) ; (b) hormone therapy in the last three months (Mann–Whitney U (exact method), $p = 0.071$); (c) oral contraception in the last three months (Mann–Whitney U (exact method), $p = 0.235$); (d) medication in the last week (Mann–Whitney U (exact method), $p = 0.657$); (e) sport/ recreation, last two days (before surgery) (Mann–Whitney U (exact method), $p = 0.167$). Post hoc tests were not performed. Outliers are shown as circles.

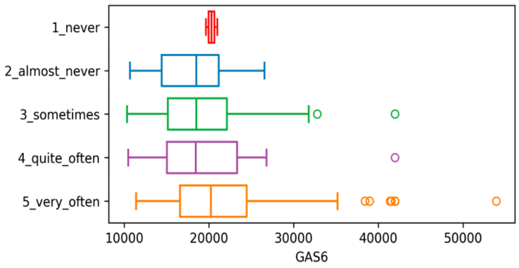

(**a**)

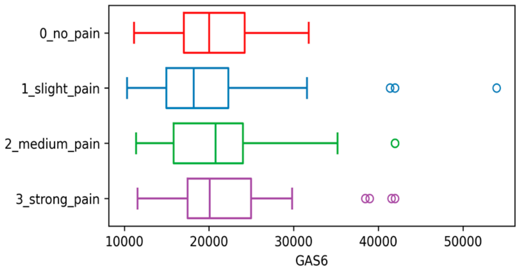

(**b**)

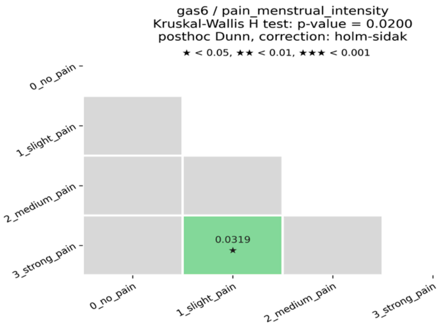

(**c**)

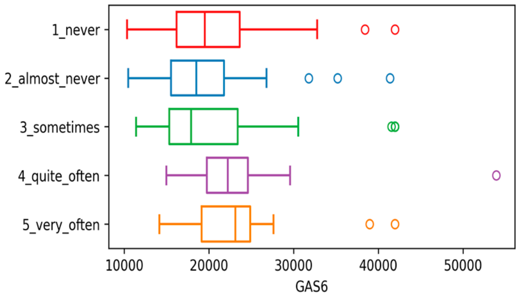

(**d**)

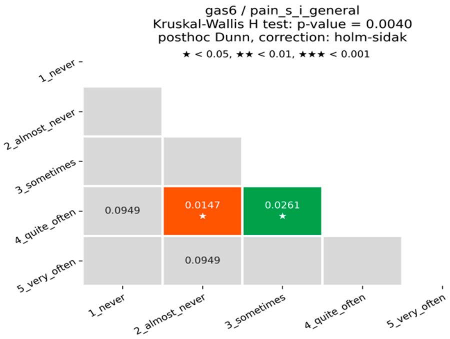

(**e**)

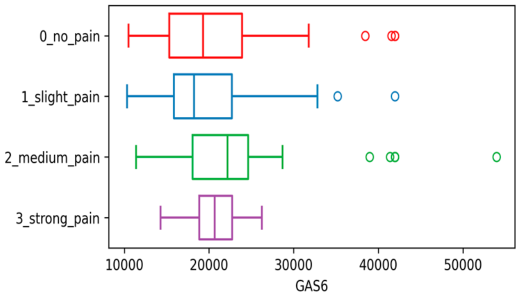

(**f**)

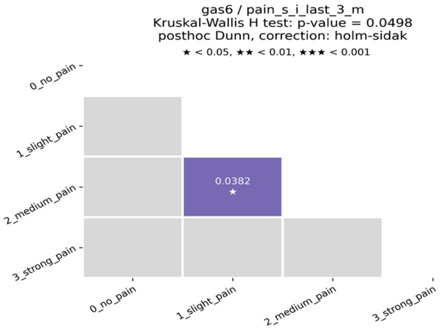

(**g**)

Supplementary Figure S4. GAS6 concentration (pg/ml) stratified by frequency of dysmenorrhoea, intensity of dysmenorrhoea, dyspareunia in general and dyspareunia in the last three months before surgery. Boxplot showing the comparison between GAS6 and (a) the patient's dysmenorrhoea frequency (Kruskal–Wallis H-test, p = 0.1143); (b) dysmenorrhoea intensity (Kruskal–Wallis H-test, p = 0.0200. (c) The Dunn post hoc test with Holm–Sidak correction showing differences between the groups with mild and

severe pain (p = 0.0319); (d) dyspareunia in general (Kruskal–Wallis H-test, p = 0.0040); (e) the Dunn post hoc test with the Holm–Sidak correction method showing differences between the groups of almost never and quite often (p = 0.0147) and sometimes and quite often (p = 0.0261); (f) dyspareunia (last three months) (Kruskal–Wallis H-test, p = 0.0498); (g) the Dunn post hoc test with the Holm–Sidak correction method showing differences between the groups with mild and moderate pain (p = 0.0382). Outliers are shown as circles.

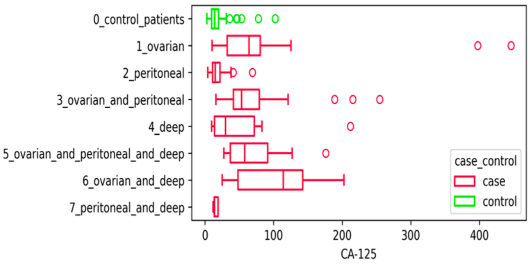

(a)

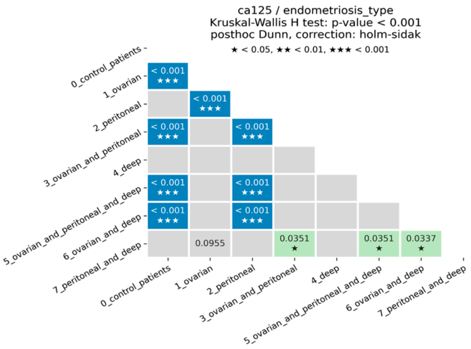

(b)

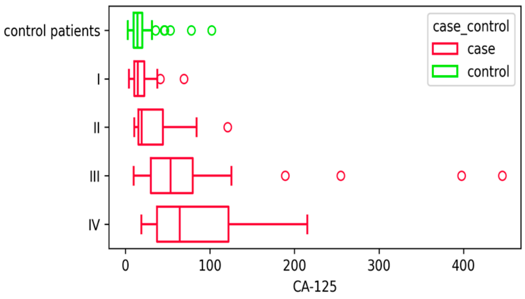

(c)

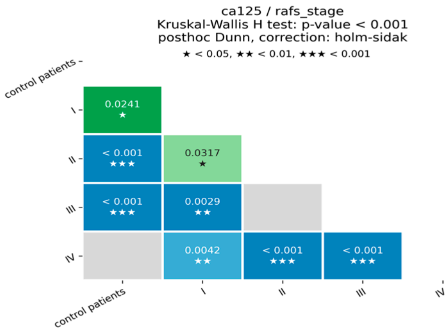

(d)

Supplementary Figure S5. CA-125 concentration by stage and type of endometriosis of the patient. Boxplot with a comparison between CA-125 and the patients' endometriosis types: (a) (Kruskal–Wallis H-test, p < 0.001); (b) Dunn post hoc test with Holm–Sidak correction method showing differences between groups; (c) rAFS stages of endometriosis (Kruskal–Wallis H-test, p < 0.001); (d) Dunn post hoc test with Holm–Sidak correction method showing differences between groups. Outliers are shown as circles.

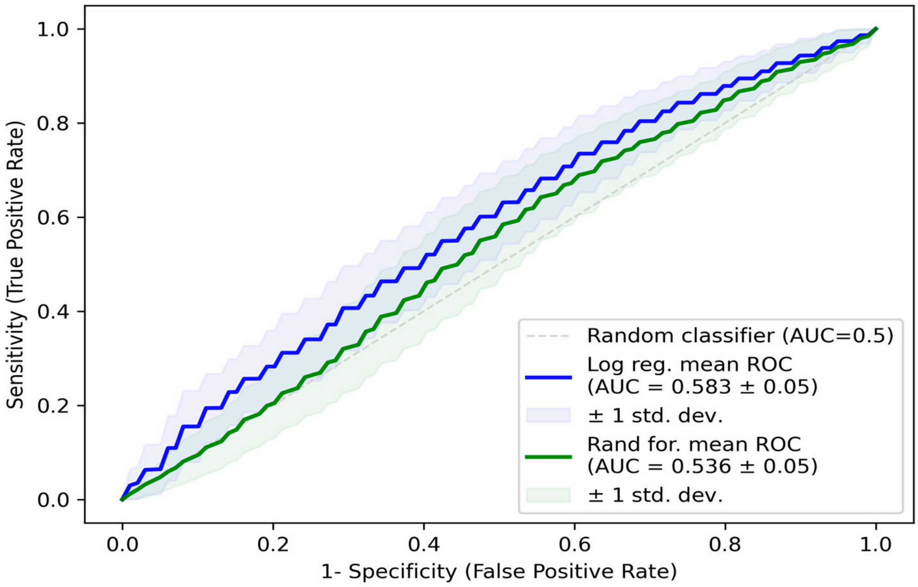

Supplementary Figure S6. GAS6 receiver operating characteristic (ROC) curve for logistic regression and random forest models. Logistic regression models with a single variable GAS6 had a mean AUC of 0.583 ± 0.05, a mean sensitivity of 96 ± 6 % and a mean specificity of 5 ± 7 %. GAS6 random forest models had a mean AUC of 0.536 ± 0.05 with a mean sensitivity of 63 ± 7 % and a mean specificity of 46 ± 8 %.

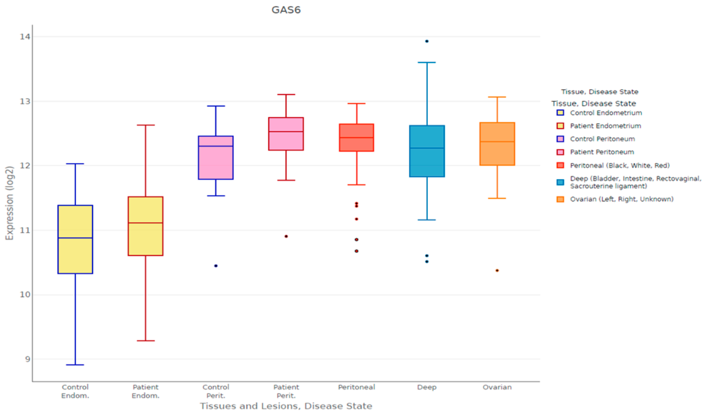

(**a**)

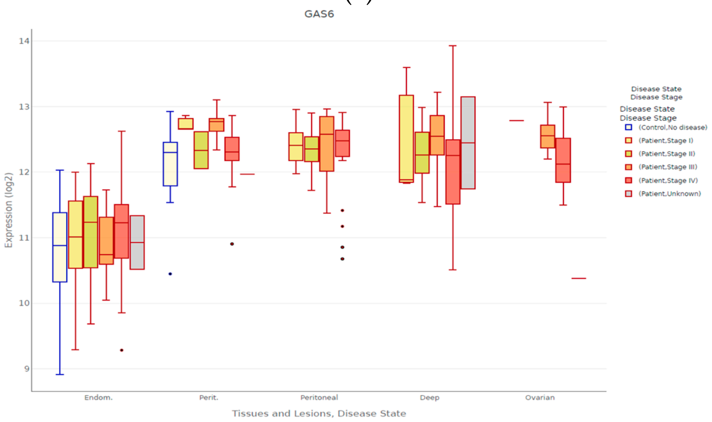

(**b**)

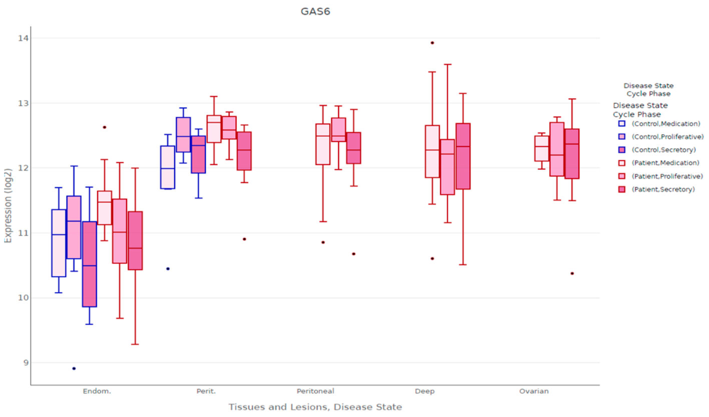

(c)

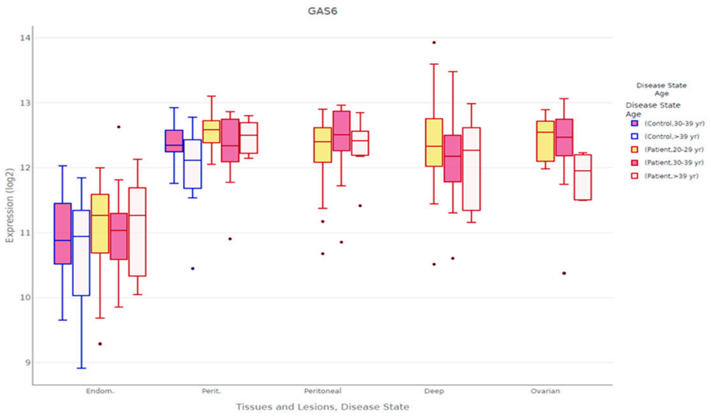

(d)

Supplementary Figure S7. Expression of GAS6 in collected tissue samples from patients with endometriosis (n=115) and controls (n=53) from the Endomet database. https://endometdb.utu.fi/gene_analysis/ [1]. GAS6 was upregulated in the peritoneum compared to normal endometrium in both controls and endometriosis patients (a). It was also upregulated in ectopic tissues of peritoneal, ovarian and deep endometriosis patients compared to their eutopic tissues (a). There were no differences in the expression of GAS6 in ectopic tissue from patients with all three types of endometriosis in relation to different rAFS stages (I-IV) (b), menstrual phases (c) or patient age (d).

References

[1] Gabriel M, Fey V, Heinosalo T et al. A relational database to identify differentially expressed genes in the endometrium and endometriosis lesions. *Sci Data*. 2020;**7**: 284.

Supplementary Table S1. **Computed logistic regression models.**

(a) Output of Best 1-variable models:

```
Best 1-variable models: log. reg., sort: auc_avg, 300 iterations
avg AUC ± sd        avg AIC ± sd        avg sensiti. %    avg specifi. %    n (case:control)|% (ca.:co.)
~~~~~~~~~~~~~~~~~~~~~~~~~~~~~~~~~~~~~~~~~~~~~~~~~~~~~~~~~~~~~~~~~~~~~~~~~~~~~~~~~~~~~~~~~~~~
ca125(0.0 ± 0.0)
0.745 ± 0.04    217.74 ± 6.81    66 ± 7    67 ± 7    284 (168:116|59%:41%)
pain_menstrual_score(0.004 ± 0.01)
0.638 ± 0.05    250.09 ± 3.54    81 ± 6    35 ± 6    284 (168:116|59%:41%)
pain_menstrual_frequency(0.006 ± 0.01)
0.628 ± 0.05    251.08 ± 3.61    77 ± 10    35 ± 8    284 (168:116|59%:41%)
pain_menstrual_intensity(0.027 ± 0.04)
0.610 ± 0.04    254.52 ± 2.91    91 ± 3    18 ± 6    284 (168:116|59%:41%)
pain_s_i_score(0.036 ± 0.05)
0.605 ± 0.05    254.97 ± 2.72    86 ± 12    19 ± 16    284 (168:116|59%:41%)
pain_s_i_last_3_m(0.032 ± 0.05)
0.595 ± 0.05    254.66 ± 2.91    79 ± 18    27 ± 24    284 (168:116|59%:41%)
pain_pelvic(0.031 ± 0.05)
0.587 ± 0.04    254.41 ± 3.16    99 ± 9    1 ± 9    284 (168:116|59%:41%)
gas6(0.163 ± 0.16)
0.583 ± 0.05    258.17 ± 2.09    96 ± 6    5 ± 7    284 (168:116|59%:41%)
pain_s_i_general(0.062 ± 0.07)
0.579 ± 0.04    256.50 ± 2.17    85 ± 14    16 ± 15    284 (168:116|59%:41%)
bmi(0.096 ± 0.11)
0.574 ± 0.05    257.17 ± 2.30    91 ± 5    13 ± 5    284 (168:116|59%:41%)
```

(b) Output of Best 2-variable models:

```
Best 2-variable models: log. reg., sort: auc_avg, 300 iterations
avg AUC ± sd        avg AIC ± sd        avg sensiti. %    avg specifi. %    n (case:control)|% (ca.:co.)
~~~~~~~~~~~~~~~~~~~~~~~~~~~~~~~~~~~~~~~~~~~~~~~~~~~~~~~~~~~~~~~~~~~~~~~~~~~~~~~~~~~~~~~~~~~~
pain_menstrual_frequency(0.048 ± 0.06), ca125(0.0 ± 0.0)
0.766 ± 0.04    214.37 ± 7.18    74 ± 7    63 ± 9    284 (168:116|59%:41%)
pain_s_i_score(0.054 ± 0.08), ca125(0.0 ± 0.0)
0.762 ± 0.04    214.38 ± 7.19    71 ± 7    66 ± 7    284 (168:116|59%:41%)
pain_menstrual_score(0.078 ± 0.09), ca125(0.0 ± 0.0)
0.759 ± 0.04    215.40 ± 6.98    71 ± 7    64 ± 9    284 (168:116|59%:41%)
pain_s_i_general(0.074 ± 0.09), ca125(0.0 ± 0.0)
0.759 ± 0.04    215.19 ± 7.16    70 ± 6    64 ± 7    284 (168:116|59%:41%)
bmi(0.112 ± 0.11), ca125(0.0 ± 0.0)
0.754 ± 0.04    216.02 ± 6.99    69 ± 7    63 ± 9    284 (168:116|59%:41%)
pain_s_i_last_3_m(0.104 ± 0.13), ca125(0.0 ± 0.0)
0.752 ± 0.04    215.88 ± 7.03    70 ± 7    61 ± 8    284 (168:116|59%:41%)
pain_menstrual_intensity(0.226 ± 0.19), ca125(0.0 ± 0.0)
0.752 ± 0.04    217.58 ± 6.95    68 ± 7    66 ± 7    284 (168:116|59%:41%)
irreg_cycles(0.235 ± 0.2), ca125(0.0 ± 0.0)
0.751 ± 0.04    217.13 ± 6.77    68 ± 8    64 ± 7    284 (168:116|59%:41%)
sport_last_2_days(0.203 ± 0.2), ca125(0.0 ± 0.0)
0.749 ± 0.04    217.19 ± 6.82    67 ± 7    65 ± 8    284 (168:116|59%:41%)
medicines_last_week(0.223 ± 0.21), ca125(0.0 ± 0.0)
0.745 ± 0.04    217.30 ± 7.01    68 ± 7    65 ± 8    284 (168:116|59%:41%)
```

(c) Output of Best 2-variable models excluding CA-125 :

```
Best 2-variable models: log. reg., sort: auc_avg, 300 iterations
avg AUC ± sd        avg AIC ± sd        avg sensiti. %    avg specifi. %    n (case:control)|% (ca.:co.)
~~~~~~~~~~~~~~~~~~~~~~~~~~~~~~~~~~~~~~~~~~~~~~~~~~~~~~~~~~~~~~~~~~~~~~~~~~~~~~~~~~~~~~~~~~~~~~~
bmi(0.05 ± 0.07), pain_menstrual_score(0.002 ± 0.01)
0.655 ± 0.05    246.48 ± 4.41    80 ± 6    41 ± 6    284 (168:116|59%:41%)
pain_pelvic(0.149 ± 0.16), pain_menstrual_frequency(0.029 ± 0.04)
0.645 ± 0.05    249.83 ± 4.37    78 ± 5    36 ± 7    284 (168:116|59%:41%)
pain_pelvic(0.2 ± 0.2), pain_menstrual_score(0.025 ± 0.05)
0.645 ± 0.05    249.47 ± 4.14    77 ± 8    36 ± 7    284 (168:116|59%:41%)
alcohol_4_2_to_3_times_a_week(0.455 ± 0.24), pain_menstrual_score(0.004 ± 0.01)
0.643 ± 0.05    250.63 ± 3.78    80 ± 7    35 ± 6    284 (168:116|59%:41%)
medicines_last_week(0.178 ± 0.19), pain_menstrual_score(0.007 ± 0.01)
0.642 ± 0.05    249.10 ± 4.03    81 ± 7    36 ± 7    284 (168:116|59%:41%)
pain_menstrual_frequency(0.031 ± 0.05), pain_s_i_score(0.194 ± 0.18)
0.642 ± 0.05    250.43 ± 3.92    79 ± 7    31 ± 7    284 (168:116|59%:41%)
pain_menstrual_score(0.025 ± 0.04), pain_s_i_score(0.25 ± 0.22)
0.641 ± 0.04    249.93 ± 3.78    80 ± 6    33 ± 7    284 (168:116|59%:41%)
pain_menstrual_frequency(0.003 ± 0.01), bmi(0.051 ± 0.07)
0.641 ± 0.05    247.58 ± 4.46    83 ± 7    38 ± 6    284 (168:116|59%:41%)
pain_menstrual_frequency(0.011 ± 0.02), gas6(0.305 ± 0.23)
0.641 ± 0.05    251.28 ± 4.05    80 ± 7    34 ± 6    284 (168:116|59%:41%)
pain_s_i_last_3_m(0.191 ± 0.18), pain_menstrual_score(0.022 ± 0.04)
0.640 ± 0.04    249.38 ± 3.96    79 ± 6    36 ± 7    284 (168:116|59%:41%)
```

(d) Output of Best 2-variable models with GAS6 as fixed predictor:

```
Best 2-variable models: log. reg., sort: auc_avg, 300 iterations
avg AUC ± sd        avg AIC ± sd        avg sensiti. %    avg specifi. %    n (case:control)|% (ca.:co.)
~~~~~~~~~~~~~~~~~~~~~~~~~~~~~~~~~~~~~~~~~~~~~~~~~~~~~~~~~~~~~~~~~~~~~~~~~~~~~~~~~~~~~~~~~~~~~~~
gas6(0.393 ± 0.24), ca125(0.0 ± 0.0)
0.745 ± 0.04    218.52 ± 6.81    66 ± 7    68 ± 7    284 (168:116|59%:41%)
pain_menstrual_frequency(0.011 ± 0.02), gas6(0.305 ± 0.23)
0.641 ± 0.05    251.28 ± 4.05    80 ± 7    34 ± 6    284 (168:116|59%:41%)
pain_menstrual_score(0.006 ± 0.01), gas6(0.299 ± 0.23)
0.634 ± 0.05    250.25 ± 3.94    81 ± 6    38 ± 6    284 (168:116|59%:41%)
pain_menstrual_intensity(0.04 ± 0.05), gas6(0.255 ± 0.21)
0.620 ± 0.05    254.33 ± 3.43    87 ± 7    26 ± 9    284 (168:116|59%:41%)
pain_pelvic(0.032 ± 0.05), gas6(0.169 ± 0.16)
0.619 ± 0.05    253.49 ± 3.74    81 ± 11    28 ± 12    284 (168:116|59%:41%)
pain_s_i_last_3_m(0.045 ± 0.06), gas6(0.232 ± 0.2)
0.606 ± 0.05    254.33 ± 3.22    81 ± 10    28 ± 9    284 (168:116|59%:41%)
bmi(0.063 ± 0.08), gas6(0.103 ± 0.11)
0.604 ± 0.05    255.11 ± 3.10    84 ± 7    20 ± 6    284 (168:116|59%:41%)
pain_s_i_score(0.052 ± 0.07), gas6(0.227 ± 0.2)
0.599 ± 0.05    254.62 ± 3.01    83 ± 8    23 ± 9    284 (168:116|59%:41%)
medicines_last_week(0.102 ± 0.13), gas6(0.16 ± 0.16)
0.597 ± 0.05    256.01 ± 3.18    87 ± 10    22 ± 11    284 (168:116|59%:41%)
sport_last_2_days(0.346 ± 0.25), gas6(0.189 ± 0.18)
0.576 ± 0.05    258.67 ± 2.48    94 ± 7    10 ± 10    284 (168:116|59%:41%)
```

(e) Output of Best 3-variable models:

Best 3-variable models: log. reg., sort: auc_avg, 300 iterations

avg AUC ± sd avg AIC ± sd avg sensiti. % avg specifi. % n (case:control)|% (ca.:co.)

~~~~~~

sport_last_2_days(0.144 ± 0.16), pain_menstrual_score(0.055 ± 0.07), ca125(0.0 ± 0.0)

0.767 ± 0.04 214.08 ± 7.05 73 ± 7 66 ± 7 284 (168:116|59%:41%)

sport_last_2_days(0.18 ± 0.18), pain_s_i_general(0.066 ± 0.09), ca125(0.0 ± 0.0)

0.765 ± 0.04 214.33 ± 7.19 71 ± 7 65 ± 7 284 (168:116|59%:41%)

menstrual_phase_OHC(0.656 ± 0.23), pain_menstrual_frequency(0.048 ± 0.06), ca125(0.0 ± 0.0)

0.762 ± 0.04 216.00 ± 7.19 74 ± 7 62 ± 9 284 (168:116|59%:41%)

menstrual_phase_proliferative(0.672 ± 0.23), pain_menstrual_frequency(0.048 ± 0.06), ca125(0.0 ± 0.0)

0.760 ± 0.04 216.03 ± 7.25 74 ± 7 63 ± 9 284 (168:116|59%:41%)

horm_therapy_last_3_m(0.601 ± 0.24), pain_menstrual_frequency(0.051 ± 0.06), ca125(0.0 ± 0.0)

0.759 ± 0.04 215.90 ± 7.22 74 ± 7 63 ± 8 284 (168:116|59%:41%)

alcohol_1_never(0.632 ± 0.23), pain_menstrual_frequency(0.051 ± 0.06), ca125(0.0 ± 0.0)

0.759 ± 0.04 215.96 ± 7.24 73 ± 7 62 ± 9 284 (168:116|59%:41%)

smoking_2_smoker(0.655 ± 0.22), pain_menstrual_frequency(0.059 ± 0.08), ca125(0.0 ± 0.0)

0.759 ± 0.04 216.03 ± 7.22 73 ± 7 62 ± 9 284 (168:116|59%:41%)

sport_last_2_days(0.166 ± 0.17), pain_s_i_last_3_m(0.086 ± 0.11), ca125(0.0 ± 0.0)

0.758 ± 0.04 214.87 ± 7.12 71 ± 7 63 ± 8 284 (168:116|59%:41%)

menstrual_phase_proliferative(0.614 ± 0.24), pain_s_i_score(0.05 ± 0.07), ca125(0.0 ± 0.0)

0.758 ± 0.04 215.89 ± 7.26 71 ± 7 66 ± 8 284 (168:116|59%:41%)

medicines_last_week(0.275 ± 0.24), pain_menstrual_score(0.095 ± 0.11), ca125(0.0 ± 0.0)

0.758 ± 0.04 215.34 ± 7.17 72 ± 7 66 ± 8 284 (168:116|59%:41%)

Models that did not converge were discarded.
~~~~~~
